# The undergraduate external examination process and the perceptions of examiners at the physiotherapy departments in South Africa

**DOI:** 10.4102/sajp.v82i2.2305

**Published:** 2026-06-26

**Authors:** Mokgobadibe V. Ntsiea, Karien Mostert, Adnil Titus, Conran Joseph, Shamila Manie, Heleen van Wyk, Muziwakhe Tshabalala, Verusia Chetty

**Affiliations:** 1 Department of Physiotherapy, Faculty of Health Sciences, University of the Witwatersrand, Johannesburg, South Africa; 2 Department of Physiotherapy, Faculty of Health Sciences, University of Pretoria, Pretoria, South Africa; 3 Division of Physiotherapy, Department of Health and Rehabilitation Science, Stellenbosch University, Cape Town, South Africa; 4 Department of Physiotherapy, Faculty of Health and Rehabilitation Science, University of Cape Town, Cape Town, South Africa; 5 Department of Physiotherapy, Faculty of Health Sciences, University of the Free State, Bloemfontein, South Africa; 6 Department of Physiotherapy, Faculty of Health Care Sciences, Sefako Makgatho Health Sciences University, Pretoria, South Africa; 7 Department of Physiotherapy, College of Health Sciences, University of KwaZulu-Natal, Durban, South Africa

**Keywords:** external examination, university examination, physiotherapy education, assessment practice, assessment variability

## Abstract

**Background:**

Variations in external examination processes across South African universities may challenge the validity of physiotherapy assessments. Our study explores external examiners’ understanding of moderation and academic standards.

**Objectives:**

This study aimed to describe the examination processes and external examiners’ perceptions of academic standards in South African physiotherapy programmes.

**Method:**

A descriptive cross-sectional survey using a self-administered questionnaire sent to all eight universities that offer a physiotherapy degree in South Africa. The total population size was 103.

**Results:**

Practices include appointing the same external examiner for up to 2 years (*n* = 4), using the same examiner for reassessments (*n* = 3), and assigning examiners across all modules regardless of expertise (*n* = 2). Five institutions allocate clinical blocks specifically, and four expose students to all clinical areas. External examiners assess all third- and fourth-year students at five institutions and receive procedural orientation via written guidelines (*n* = 5). Feedback is typically shared orally (*n* = 5) or in a university-formatted written report (*n* = 3). A 10% mark difference is acceptable at two institutions; others lack clear criteria. Internal and external marks carry equal weight, and two universities apply sub minima for clinical safety and effectiveness. Moderation samples span high to low scoring scripts (*n* = 4). Contingency plans for examiner cancellations are in place at all institutions. Indicators of good performance include evidence-based practice (88%), alignment with departmental outcomes (71%), and meeting examiners’ assessment expectations (50%).

**Conclusion:**

Clarification of the external examiner’s role is needed in the absence of formal guidelines from governing bodies.

**Clinical implications:**

Findings can guide consistent competency standards and also support the Health Professions Council in evaluating physiotherapy training institutions.

## Introduction

Many differences exist between countries and universities in the external examination process.

In some countries or universities, academic staff have considerable autonomy in how they set and grade an examination, and some countries implement a complex system that creates a common procedural standard across the country or universities (Clements 2005). General standardised moderation procedures for most universities within countries that have a common procedural standard are as follows: an academic sets an examination and memorandum or model answers that are sent to a senior academic and an external examiner at another university (Smith & Farwell 2011). The external examiner comments about the suitability of the examination, and the academic would modify the examination paper and procedure if required after input from internal and external examiner. After the completion of the examination, the external examiner samples the students’ work and comments on performance and results. Generally, the external examiner is responsible for the quality of the examination process (Smith & Farwell 2011).

The role of external examiners is primarily to act as moderators, ensuring the quality, fairness, and consistency of assessment practices (Council of Higher Education 2013). According to the South African Higher Education Quality Committee of the Council of Higher Education, a moderator is a person, apart from the examiner, who is appointed by the institution to be responsible for ensuring the standard of the examination and its accompanying marking framework and response exemplars, and for marking a representative sample of examination responses. They do not carry out the marking of individual students’ assessed work but moderate the marking carried out by internal examiners. This process involves viewing student work (whether reading scripts or viewing live or recorded performances), usually on a sample basis. This enables the external examiner to form a view as to whether the internal marking has been carried out to rigorously judge students’ performance against the university’s standards, and against the national and professional body’s standards, including adherence to fairness and consistency. The use of moderators, when diligently practised, has considerable value in establishing and maintaining standards for higher education. However, their main limitation is that they are institutionally controlled and localised (Council of Higher Education 2013).

Each institution of higher learning has autonomy but follows guidelines by the higher education qualifications authorities and those of professional bodies within their countries. Most institutions follow the guidelines of moderation of a minimum (Min) of 10 assessment items or 10% of assessment items (whichever is greater) for internal and for external moderation (Study Group Australia Higher Education Division 2018). The persons selected for the role of moderator must be able to detect errors, discrepancies or lack of skill and ability in the marking process of a particular module. A moderator should understand the system that the institution uses in order to execute their duties successfully. Some universities have an internal and external moderation system, while some only have an external moderation system. Some institutions do external moderation after releasing grades with the aim of getting moderator feedback to improve the following year, and on the other hand other institutions do moderation before grades are released with the aim of changing current marks in addition to making changes for the following years based on external moderator feedback (Study Group Australia Higher Education Division 2018). Some institutions have a standard moderation tool to report on the moderation process, and others leave this up to the moderator and thus may not get reports based on the same items over years of moderation or even from different moderators within the same year. When the external examiner reports on different components each year, it does not give them an opportunity to monitor and respond to changes over time (Gabieba, Katlego & Felicity 2025).

A moderator may not be familiar with the institution’s moderation method that is used if they do not get an information pack or briefing prior to serving as a moderator. Different moderation methods that some institutions may use include: *Blind moderation* (second marker does not see the first marker’s results); *Double moderation* (two markers mark the same piece of work and comment on the original piece of work are seen by second marker) and *Exchange moderation* (two markers exchange two pieces of work) (Study Group Australia Higher Education Division 2018).

In a study by Bloxham and Price (2014) on external examiners’ understanding and use of academic standards, the examiners interviewed had contrasting views about their role with regard to standards: at one extreme, examiners import their own personal standards that may or may not be aligned with national standards, and use these alone to judge the quality of a programme’s assessment; at the other extreme, examiners defer to the awarding institution’s standards and focus only on assessment procedures. In some instances, the examiners did not see guaranteeing standards as part of their role.

It is thus necessary to know the external examiners’ understanding of the moderation process and their understanding and use of academic standards. The researchers are not aware of any study conducted in a South African setting, especially the physiotherapy field, about the external examination process and how the external examiners perceive the examination standards and processes. The question is whether the external examiners’ advice is based on their understanding of the Higher Education Framework, personal or own institution experiences or policies of the Health Professions Council of South Africa (HPCSA). The responsibility for clarifying the role of the examiner with regard to standards lies with individual institutions (Council of Higher Education 2013). Another question is what informs the institution’s decisions about the role they wish an examiner to take and to what extent they want examiners to draw on wider disciplinary standards and reference points or whether they are satisfied with independent oversight of assessment procedures. Our study was conducted with the following aims: To describe the examination procedures in the physiotherapy programme at the South African universities and how those who have served as external examiners perceive the examination standards and process.

## Research methods and design

### Study design

A descriptive cross-sectional survey was carried out using a self-administered questionnaire.

### Study population

To describe the examination procedures of third- and fourth-year clinical, theoretical and research modules in physiotherapy courses at the eight South African universities, which is a total number of institutions that have a physiotherapy course in the country. The focus was on third and fourth years because these are the clinical years of study, which cover both theory and clinical examinations. The study population was physiotherapy academics in South Africa who have served as external examiners. There are about 103 academics based on university webpages at the eight universities, but there is no database that shows how many of these academics served as external examiners. The source of data was the third- and fourth-year module coordinators. Each head of department was asked to refer the research team to the relevant coordinator. As the population is small, all the universities in South Africa that teach the undergraduate physiotherapy degree were considered for our study.

To ascertain the profile of physiotherapy academics and how those who have served as external examiners perceive the examination standards and processes, all full-time academics at each institution, who have already acted as an external examiner either locally or internationally at the time of the study, were eligible to participate.

### Instrumentation

A self-administered questionnaire was developed by the researchers using physiotherapy education and general examination-related literature. Questions were formulated in relation to the study objectives, and content was validated through input from physiotherapy academics.

The questionnaire for staff members had questions about age, gender, year graduated, highest qualification, years in academia, number of times acted as external examiner, and area of interest.

In addition, class coordinators completed section about the characteristics of the department, such as number of staff members per student, administrative support for logistical arrangements of external examiners’ accommodation and transport, the criteria used to choose external examiners, the specific clinical block allocation structure for third and fourth year, assessment procedures (the process of moderation of clinical assessments), the process of moderation of theory examinations, the process of moderation of practical examinations, the process of moderation of research and existing general examination contingency plans (e.g. because of unforeseen circumstances such as student riots and pandemics).

### Data collection

To describe the examination procedures of third- and fourth-year clinical, theoretical and research modules in physiotherapy courses at eight South African universities, an introductory email was sent to the eight universities explaining the purpose of the study, the number of questions and the time demanded to complete the questionnaire and that participation was optional and anonymous, and that no financial incentives would be offered. Study data were collected and managed using REDCap electronic data capture tools hosted at the University of the Witwatersrand (Harris et al. 2009). A questionnaire was shared with the class coordinators who were given 2 weeks to complete the questionnaire. An automated REDCap reminder email was sent after weeks 1 and 2.

To ascertain the profile of physiotherapy academics and how those who have served as external examiners perceive the examination standards and process, physiotherapy heads of departments (HODs) at each of the eight universities were contacted electronically to participate in our study. The primary investigator provided background to the study as well as the ethics approval letter for the research project in this initial communiqué. Once approval from the HODs had been obtained, full-time academic staff were emailed with a request to complete an electronic questionnaire. Staff members who volunteered to complete the questionnaire were given 1 week to submit their completed questionnaire. Electronic reminders were sent out weekly for a duration of 4 weeks to each staff member after the initial recruitment email.

### Data analysis

Descriptive data analysis was applied: Frequencies and percentages for categorical data and means, and standard deviations (s.d.) with ranges for continuous data.

### Ethical considerations

Ethical clearance to conduct this study was obtained from the University of the Witwatersrand Human Research Ethics Committee (Medical) (No. M180287).

## Results

### The profile of the study participants and how they perceived the examination standards and processes

Twenty-eight academics from South African physiotherapy departments responded to the questionnaire. The average age of the study participants was 39.4 years (Min 27, maximum [Max] 56) and mostly females (*n* = 18, 64.3%). Most of the academics had been qualified as physiotherapists for more than 10 years and have been working as academics for less than 10 years (Table 1).

**TABLE 1:** Number of years qualified as a physiotherapist and working as an academic (*N* = 28).

Number of years	Years qualified as a physiotherapist		Years working as a physiotherapy academic	
	*n*	%	*n*	%
1–5	2	7	5	18
> 5–10	2	7	14	50
> 10–15	9	32	2	7
> 15–20	7	25	6	21
> 20–30	8	29	1	4
> 30	0	0	0	0

The distribution of participants by their qualifications, external examination history, year of study they taught and what they taught is presented in Table 2. Twenty-seven of the study participants had post-graduate qualifications; most of them had a Master of Science in Education Physiotherapy (*n* = 20, 74%), and 24 (86%) had served as physiotherapy undergraduate external examiners. Most of the physiotherapy academics who participated in the study taught in the second (*n* = 23, 82%) and third year (*n* = 24, 86%) of the 4-year Physiotherapy degree, and many of them taught research (*n* = 13, 46%) followed by adult neurology and musculoskeletal conditions (*n* = 10, 36% each).

**TABLE 2:** Study participants by qualifications, examination, year of study taught and what they teach (*N* = 28).

Participants’ characteristics	*n*	%
**Additional qualifications**		
MSc Physiotherapy	20	74
PhD	12	44
MPH	1	4
**Year of study they teach**		
First	11	39
Second	23	82
Third	24	86
Fourth	19	68
**What they teach**		
General paediatrics	0	0
Public health/Community	8	29
Paediatrics neurology	1	4
Adult neurology	10	36
Cardiopulmonary	8	29
Orthopaedics	4	14
Neuro-musculoskeletal	10	36
Sports	2	7
Orthopaedic manipulative therapy	4	14
Research methodology	13	46
**Served as an external examiner (UG, PT)**		
Yes	24	86
No	4	14

MPH, Master of Public Health; MSc, Master of Science in Education; PhD, Doctor of Philosophy; PT, physiotherapy; UG, undergraduate.

### External examiner roles and responsibilities

The majority of the study participants indicated that when they are invited to serve as external examiners for clinical and practical examinations, they expect information about the marksheets and guiding rubrics to be used during the examination, what the primary focus of the examination is, and marking procedures (Table 3). These expectations are similar for the period before and on the day the examination commences, except information about course outlines and curricula, which is at least expected by the first day of examination.

**TABLE 3:** Information expected by external examiners before and on examination day when invited to examine a clinical or practical examination.

Before examination day (*n* = 24)	*n*	%	On examination day (*n* = 21)	*n*	%
Mark sheets and grading rubrics to be used during the examination	23	96	What the primary focus is on (e.g. assessment and/or treatment)	21	100
What the primary focus is on (e.g. assessment or treatment or both)	22	92	Mark sheets/grading rubrics to be used during the examination	20	95
Marking procedures (e.g. whether examiners discuss marks, whether subminima apply)	21	88	Marking procedures (e.g. whether examiners discuss marks, whether subminima apply)	20	95
Examination timetable	20	83	Details about examination logistics and venue(s) and areas	19	91
Details about examination logistics and venue(s)/areas	19	79	How significant differences internal and external marks are dealt with	18	86
Whether feedback required after examination is about students’ performance and/or module	19	79	Examination timetable	15	71
What is considered a significantly difference in marks between examiners	17	71	What is considered a significant difference in marks between examiners	15	71
What to do when internal and external marks differ significantly	17	71	What is considered below average, above average, very good, excellent etc. performance	15	71
What is considered below average, above average, very good and excellent performance	17	71	Whether feedback required after examination is about students’ performance and/or module or programme	11	52
Course outlines and curriculum	16	67	Whether institution provides additional solicited views (what they want external examiner to assess and comment about)	6	29
Whether department requires additional solicited views (e.g. wanting external examiner to play advocacy role – justify for posts/equipment)	10	42	Whether department requires additional solicited views (e.g. wanting external examiner to play advocacy role – motivate for posts/equipment etc.)	6	29
Whether institution provides additional solicited views (what they want external examiner to assess and comment about)	9	38	Course outlines and Curriculum	3	14

Many of the study participants indicated that when they are invited to serve as external examiners for theory examinations they expect the following before the examination: Question paper (*n* = 24, 100%), Memoranda (*n* = 23, 96%), Logistics (when to expect papers and by when and they should be returned to the department) (*n* = 22, 92%), and course outline and curriculum (*n* = 19, 79%). After examinations they expect the following: Sample of answer sheets (*n* = 23, 96%), Class list with marks for entire class (*n* = 20, 83%), information about what to do when there are significant differences between examiners (*n* = 18, 75%), and what is considered significant difference in marks between examiners (*n* = 16, 67%). One person (*n* = 1, 4%) indicated that they need to see all answer sheets after examination.

### What is used as an indicator for student performance?

The HPCSA (*n* = 15, 63%), university (*n* = 13, 54%), Faculty (*n* = 5, 21%), School (*n* = 4, 17%), and Department (*n* = 17, 71%) guidelines are used to determine the standard of students’ performance. The following are considered indicators of good student performance: Evidence-based practice (*n* = 21, 88%), students’ performance in relation to departmental learning outcomes (*n* = 17, 71%): When students’ performance satisfy the external examiners’ beliefs about assessment and treatment approaches (*n* = 12, 50%), when external examiner gives the same or more marks than internal examiner (*n* = 6, 25%), students doing better than external examiner’s university students (*n* = 3, 13%), if their knowledge or clinical skills is similar to that of the external examiner (*n* = 2, 8%) and if they do what internal examiner says they were taught irrespective of external examiners’ ‘assessment philosophy’ (*n* = 1, 4%).

### External examination process at the physiotherapy departments in South Africa

The profile of the five physiotherapy departments that responded to the questionnaire about the external examination process: had on average 60 (s.d. 15, Min 22, Max 70) first-year students; 55 (s.d. 20, Min 23, Max 69) second years; 47 (s.d. 5, Min 39, Max 53) third years and 46 (s.d. 5, Min 38, Max 50) fourth years. The average number of full-day staff members per department was 12 (s.d. 1, Min 10, Max 13) with a maximum of two part-time academics. Three of the five universities indicated that they have clinical/sessional staff members. The average number of clinical staff per department is 12 (s.d. 7, Min 4, Max 18), and they work 15 h per week (s.d. 7, Min 10, Max 20). The departments had a maximum of two administrators.

Four of the five universities invite the same external examiner for a period of 2 years and one for 3 years. Three of the five universities use the same external examiners for re-assessment and repeating students, and two use the same external examiner available for all modules regardless of the examiners’ area of teaching or research expertise. All five universities have specific clinical block allocations, and four of the five had students work in all the blocks.

The components presented by the physiotherapy department that were externally moderated for the third year were Theory (*n* = 1, 33%), Practical (*n* = 1, 33%), and Clinical (*n* = 3, 100%) and for the fourth year, the components were Theory (*n* = 2, 67%), Clinical (*n* = 3, 100%), and Research (*n* = 3, 100.0%). All third and fourth years were externally examined. Orientation of external examiners regarding the procedures was performed by all five universities through written guidelines before the assessment opportunity. External examiners provided feedback after the examination opportunity orally during examiners’ meetings (*n* = 5, 100%), and with a written report using their own format (*n* = 2, 40%) or a written report using a template provided by the department (*n* = 3, 60%).

### The process of moderation of clinical assessments

At three of the five universities, the external examiners do not moderate all clinical blocks. All five universities had clinical blocks externally examined at the end of the third- and fourth-year examinations. Elements of the clinical blocks that were externally examined were mainly both treatment and assessment in the fourth year, with one university that externally moderates only the treatment in the third year. Four of the five universities used a combination of formative and summative assessment methods for both theory and clinical examinations, with one university using only a summative method. Four of the five universities have supplementary and re-examination opportunities, and they use external examiners during the supplementary/re-examination, except one university that only moderated the supplementary and re-examination internally.

Four of the universities require examiners to provide a separate mark and take an average of all examiners, and one university allows examiners to discuss and decide on a single mark. In cases where there were major differences in marks allocated, examiners were requested to discuss and submit marks in the same category, such as fail or pass (*n* = 2, 40%). If examiners did not agree (e.g. pass and fail), an average mark was used to obtain a final outcome (*n* = 1, 20%). In instances where examiners did not agree about a mark (e.g. pass and fail), the members of the examiners’ meeting made the final decision (*n* = 1, 20%). If there were differences in marks allocated by the external and internal examiners, a difference of 10% was considered acceptable. If there were differences between examiner marks, all five universities considered both internal and external examiner marks to carry the same weight. Two of the five universities applied sub minima and they were applied for effectiveness, safety of the patient or model and safety of the therapist.

### The process of moderation of theory examinations

Three out of the five universities’ theory examination papers were moderated by an external examiner, and all five universities had papers moderated before the examination, and all answer sheets were moderated by an external examiner. Answer sheets were sampled through random selection (*n* = 1, 20%). In addition, four universities (80%) sampled answer sheets from three categories of students: students who achieved relatively high, moderate and low marks. At one university, a four-mark discrepancy between examiners’ theory scores was considered significant, whereas two universities regarded a 10-mark difference as significant. When there was a significant difference in theory marks between internal and external examiners, they had to remark (*n* = 4, 100%) and retrieve a larger sample (*n* = 1, 25%). Two universities did not indicate what was considered a significant difference in theory marks between examiners.

Elements that were moderated by an external examiner were second-year question paper (*n* = 1, 20%), second-year answer sheets (*n* = 1, 20%), third-year question paper (*n* = 2, 40%), third-year answer sheets (*n* = 2, 40%), fourth-year question paper (*n* = 5, 100%) and fourth-year answer sheets (*n* = 5, 100%). Research components that were moderated by external examiners were the research proposal (*n* = 2, 40%), research reports (*n* = 1, 20%), research presentations (*n* = 4, 80%), poster (*n* = 1, 20%), and manuscript (*n* = 1, 20%). Two universities used summative research assessment, and three used both summative and formative formats. Additional components that were moderated together with the question papers were memoranda (*n* = 5, 100%), the weight of different levels of Bloom’s taxonomy (*n* = 1, 20%). Additional documents that accompanied the answer sheets to external examiners were course objectives and outcomes (*n* = 1, 20%), university assessment policy (*n* = 1, 20%), class lists (*n* = 2, 50%), lists with internal examiner marks (*n* = 2, 50%) and the final mark breakdown (*n* = 1, 25%).

All universities that participated in our study had contingency plans in case examinations could not continue for clinical, practical or theory. Examination contingency plans that were executed were a change in venues (*n* = 4, 80%), rescheduling for later dates within the same academic year (*n* = 4, 80%), and using internal examiners for moderation if an external examiner was no longer able to assist (*n* = 1, 20%).

## Discussion

### External examination process at the physiotherapy departments in South Africa

The majority (80%) of the universities invite the same external examiner for a period of 2 years and use the same external examiner for reassessments and repeating students (60%).

Only 40% of the universities used the same external examiner for all the modules. In the United Kingdom (UK) assessors are appointed for a period of usually 4 years (Clements 2005). Unfortunately, limited information is available on the reasons for the decisions or even to compare to other countries. Anecdotally, the reason for prolonged appointment as external examiner is linked to whether valid suggestions for improvements are observed in subsequent years. Having an examiner for a long time has the risk of leading to familiarity, which decreases oversight responsibility. There are advantages to long-term appointment in that the examiner will develop programme knowledge, and this is important for quality assurance.

Different components that were externally moderated at the participating universities included: theory and clinical components in both the third and fourth years of study, as well as the research (theory) component. All third- and fourth-year students were externally assessed in all the universities.

In terms of administrative procedures, all universities orientated external examiners before the commencement of the assessment opportunity, whereafter they were expected to provide feedback during examiners’ meeting as well as complete a written report. In the UK, most universities provide a template report, and feedback between universities and external examiners indicated that the reports tend to be similar (Bloxham & Price 2014; Clements 2005). Unfortunately, no detail on the content of the reports was asked during our research, but it would be beneficial to determine whether similar aspects are being reported on. This is also in line with the expectation of external examiners, where they can visit the department as well as complete a written report (Clements 2005). Reports and feedback are an important method for external examiners to comment on any part of the moderation, provide advice and recommend any suggestions to improve the quality and the standard of the student training.

The aforementioned is essentially the fundamental role of the external examiner, in addition to the quality control and the monitoring of the examination procedure (Clements 2005). The Australian College of Natural Medicine Pty Ltd (ACNM) 2024 indicated that a diverse range of fair, equitable, clear, meaningful, and specific assessment instruments and processes may be used as part of their assessment policy. To standardise the format of feedback and reporting in SA universities would be very beneficial to ensure that the external examination process is clear in purpose, concise and comparable across all the universities.

### The process of moderation of clinical assessments

There is consistency that all the SA universities externally examine students on treatment and assessments at the end of the third and fourth years, although not on all the blocks. Most SA universities use formative and summative assessment methods and have supplementary and re-examination and rely on external examiners. The use of formative assessments in physiotherapy is beneficial because it provides an opportunity for the student to get feedback and improve their skills on an ongoing basis for developmental purposes.

Summative assessments are also needed to assess competency, and the scores increase when the assessment is summative versus formative (Donker et al. 2025). At most South African universities, examiners award marks independently, after which a final mark is calculated as the average; in contrast, one university requires examiners to agree on a single negotiated mark. Different methods were used in the case of discrepancies, for example, discussing and awarding marks in the same percentage category, individual mark and used average, examination committee making the final decision, a difference of 10% was considered acceptable, the external examiners’ marks carry more weight. There is no single method that is considered the best; however, it is recommended that for summative assessment, two examiners should be used to assess and that judgement about the final mark should be reached by consensus between the examiners (Faherty et al. 2020). Only two SA universities applied sub minima for effectiveness and patient or therapist safety. No information is available to compare to other countries on the exact procedure during clinical assessments.

The Australian College of Natural Medicine Pty Ltd (ACNM) 2024 recommended that Clinical and Practical Subjects, where the graduate will be expected to consult and treat clients, support specific pass levels for practical and clinical subjects, as students need to acquire and maintain a high degree of skill to be effective in practice. The Australian College indicated that there should be clear marking rubrics indicating the criteria that will be used to assess a student as well as the standards for those criteria. These assessment criteria and standards must align with the subject learning outcomes. A study carried out in the UK indicated the understanding between internal and external examiners of standards and communication between examiners is of utmost importance (Bloxham & Price 2014). Most SA and UK universities agree that the external examiner’s principal role is in quality control and the monitoring of the exam procedure.

During a study carried out on the assessment of student nurses, it was also highlighted that although there were some measures in place to ensure the validity and reliability of assessments, there is a need to develop a policy on clinical learning assessment that aligns assessments with clinical learning outcomes. It is important to determine whether the clinical assessment tools used in physiotherapy clinical assessments are valid and reliable. If the reliability and validity of the tools are not available, then the quality assurance purpose is lost. The primary focus should be to improve the preparation of students for clinical learning assessment (Kayihura & Mtshali 2010) to determine readiness for clinical practice.

### The process of moderation of theory examinations

All SA universities moderated theory examination papers (before the examination) and answer sheets (after the examination) externally. This aligns well with the Health Professions Council of SA minimum standards of training (quality assurance), which specify that all exit-level module outcomes and all final year courses or modules must be externally moderated. Most of the SA universities (80%) sampled answer sheets from three categories (high, moderate and low marks). Different marks were considered as a significant difference between internal and external examiners’ marks, a mark difference of four as well as 10 was indicated, two of the SA universities did not indicate the consideration for significant difference. The process that followed the detection of the significant difference was also different, as one SA university retrieved a larger sample and 80% remarked the answer scripts. Research comprised of different aspects being assessed both summative and formative, for example, proposal (40%), reports (20%), presentations (80%), poster (20%) and manuscript (20%).

Additional components that accompanied the papers included: memoranda (100%), weight of different levels of Bloom’s taxonomy (20%), course objectives (20%), class list (50%), internal examiner’s marks (50%), as well as final mark breakdown (25%). All SA universities had contingency plans if examinations could not continue, for example, change venues (80%), reschedule in the same academic year (80%), and make use of an internal moderator if external examiners are not available (20%).

According to a report completed by Clements (2005), the external examiner views the scripts for accuracy, adherence to the curriculum as well as the quality of the marking and can determine whether there are differences or variations in poor, good and very good student performance. The details of the exact process are unknown.

Suggestions of the information that should be included in external examiner’s feedback for theoretical, clinical, practical and or research are: feedback regarding the standards, for example, does the standard adhere to the minimum requirements of the national standards, or benchmark to other institutions, suitability and range of the teaching and learning methods, application of evidence-based teaching and learning, alignment with the course outcomes, quality and availability of resources. Other aspects may include whether the process is fair and valid; quality of the assessment and the assessment opportunity; appropriateness of the marking grids; general impression of the assessment process and student performance. The external examiner can identify strengths and weaknesses and recommend how to improve the quality of the assessment process (Bloxham & Price 2014; Clements 2005). If an external examiner has concerns about the internal marking, the examiner could consider moderating a larger sample, re-marking or the scaling upwards or downwards of the marks for the entire cohort of work in question (Bloxham & Price 2014). The external examiners, according to input given in our study, can also benefit from having curriculum alignment documents before the examination in order to ensure applicability and appropriateness of the examination questions and processes.

It is important to realise the importance of an external examiner as we want to equip students to be the best healthcare professionals that they can be and to be advocates for our profession. External examiners can also act as mediators in the cases of internal disputes. Although these recommendations can be perceived as ‘cruel or incriminating’ if there are standardised outcomes, we will adhere to the guidelines of the HPCSA and ensure that all the students adhere to the same standards, and it is more comparable, and this can lead to more confidence in the assessment process. Additional benefits of having external examiners include the opportunity to identify mentors beyond the individual university, as well as fostering collaborations between external examiners and the educators.

Future research should be conducted to rate the priorities among academic institutions on processes, strategies and actions to include to enhance standardisation of external examination processes. In this way, the process will require and endorse feedback from all stakeholders in order to provide recommendations for the future. It would be beneficial to the universities if external examiners’ report forms could contain key components, for example, (1) the minimum expectations; (2) whether it aligns with outcomes and standards; (3) if the programme is consistent with the national standards and expectations; (4) if the student’s performance is comparable with similar programmes with which the examiner is familiar; (5) evaluation of whether the marking process is thorough and fair; (6) assessment of whether internal assessment is appropriate to the required standard; (7) assessment of whether internal assessment is appropriate to the required standard; (8) identification of strengths and weaknesses within the programme; and (9) recommendations and suggestions for programme improvement.

### The profile of the study participants

Of the 28 academics who responded, the majority were female with an average age of 39 years. Interesting to note was that the majority had been qualified as physiotherapists for more than 10 years but had been working for less than 10 years as an academic. Most participants had post-graduate qualifications. Eighty-six per cent of participants were externally examined at more than one institution. Most academics taught in the second and third year and taught research, adult neurology and musculoskeletal conditions. No other information regarding the profiles of external examiners could be found, indicating the importance of looking into our study.

## Conclusion

All third- and fourth-year students are externally examined, and the examination format is generally a combination of summative and formative. Clarification of the external examiner’s role is needed in the absence of formal guidelines from governing bodies. There is a need to clearly exemplify the role of the external examiner in physiotherapy education to ensure clarity on expectations. The findings of our study may be used as a guide to ensure consistency in the expected standard of competencies of graduates across the country. In addition, the HPCSA may use the findings as a guide when evaluating training institutions.

### Limitations of the study

The detailed contents of the external examiners’ reports were not collected to provide reasons for the choices made.

The self-developed questionnaire had content validity, and no reliability was tested.
